# 罗特西普联合罗沙司他治疗难治性骨髓增生异常肿瘤伴环状铁粒幼细胞的随机对照前瞻性单中心研究

**DOI:** 10.3760/cma.j.cn121090-20241126-00484

**Published:** 2025-07

**Authors:** 心怡 卢, 杼新 张, 紫薇 刘, 辰 杨, 苗 陈, 冰 韩

**Affiliations:** 中国医学科学院北京协和医院血液内科，北京 100730 Department of Hematology, Peking Union Medical College Hospital, Chinese Academy of Medical Science, Beijing 100730, China

**Keywords:** 骨髓增生异常肿瘤, 罗沙司他, 罗特西普, 红系反应, 前瞻性随机对照研究, Myelodysplastic neoplasms, Roxadustat, Luspatercept, Erythroid response, Prospective randomized controlled trial

## Abstract

**目的:**

观察罗沙司他联合罗特西普治疗难治性低危骨髓增生异常肿瘤（MDS）伴环状铁粒幼细胞（MDS-RS）的疗效和安全性。

**方法:**

本研究为单中心前瞻性随机对照试验，招募至少经rhEPO治疗后难治的MDS-RS患者，按照1∶2比例随机分配至罗特西普和罗沙司他联合用药组和罗特西普单药组。主要终点为治疗12周红系反应，次要终点包括治疗24周红系反应、治疗12周内脱离输血时间≥8周等。

**结果:**

共入组48例患者，联合用药组（16例）和单药组（32例）在基线人口学特征、实验室指标、IPSS-R危险度、输血负担、EPO水平和既往治疗史等方面差异均无统计学意义（*P*>0.05）。在相似的罗特西普治疗剂量和随访时间内，治疗12、24周时，联合用药组和单药组获得红系反应、脱离输血依赖情况及其他指标变化方面差异均无统计学意义（*P*>0.05）。两组的不良反应发生率相近（*P*>0.05）。

**结论:**

罗特西普联合罗沙司他治疗难治低危MDS-RS的疗效和安全性与单用罗特西普没有显著差异。**临床试验注册**：中国医学科学院北京协和医院（K3697）

## Abstract (en)

**Objective:**

To evaluate the efficacy and safety of luspatercept combined with roxadustat in patients with refractory low-risk myelodysplastic neoplasms with ring sideroblasts（MDS-RS）patients.

**Methods:**

In this single-center, prospective, randomized controlled trial, patients with refractory MDS-RS were randomly assigned in a 1:2 ratio to receive either combination therapy（luspatercept + roxadustat）or luspatercept monotherapy. The primary endpoint was erythroid response at 12 weeks, while secondary endpoints included erythroid response at 24 weeks, achievement of transfusion independence ≥8 weeks within the first 12 weeks, and other hematologic indicators.

**Results:**

The combination therapy and monotherapy groups included 16 and 32 patients, respectively. Baseline demographic characteristics, laboratory tests, IPSS-R risk classification, transfusion burden, EPO levels, and previous treatment history were comparable between the two groups（*P*>0.05）. With similar doses of luspatercept and follow-up durations, no significant differences were observed between the groups at either 12 or 24 weeks in terms of erythroid response, transfusion independence, or other clinical indicators（all *P*-values>0.05）. The incidence of adverse events was similar in both groups（all *P*-values>0.05）.

**Conclusion:**

Luspatercept combined with roxadustat shows comparable efficacy and safety to luspatercept monotherapy in the treatment of refractory low-risk MDS-RS. **Clinical trial register**：Peking Union Medical College Hospital, Chinese Academy of Medical Science, Beijing（K3697）

骨髓增生异常肿瘤（myelodysplastic neoplasms, MDS）是一组异质性的髓系克隆性疾病，其主要特征是血细胞减少和向急性髓系白血病转化的风险增加[1]。MDS伴环状铁粒幼细胞（MDS-RS）是MDS的一种亚型，其特点是RS≥15％，或RS≥5％伴SF3B1基因突变。

重组人促红细胞生成素（rhEPO）是较低风险MDS的一种主要治疗策略，但其有效率仅为50％[2]–[3]。对于rhEPO治疗无效的患者，后续的治疗选择非常有限。有研究表明，去甲基化药物和免疫调节剂等二线治疗方式有效率低，且不能显著延长这些难治性患者的总生存时间[4]。

罗沙司他（Roxadustat）通过低氧诱导因子（HIF）通路上调转铁蛋白受体、上调转铁蛋白和下调铁蛋白水平以促进铁的吸收和转运[5]。对于低输血负担的低危MDS患者，一项正在进行的Ⅱ/Ⅲ期临床试验（NCT03263091）早期结果发现，38％的患者脱离输血依赖至少8周，58％的患者红细胞输注负担降低达50％[6]。罗特西普（Luspatercept）是一种能够促进晚期红细胞分化并抑制TGF-β信号以调节骨髓微环境，最终改善红系造血的药物[7]。MEDALIST Ⅲ期临床试验结果表明，罗特西普可显著改善EPO难治MDS患者的输血依赖[8]。国内一项多中心回顾性研究也证实，难治性低危MDS-RS患者接受罗特西普治疗后HGB明显升高、血清铁蛋白水平显著下降且安全性良好[9]。

鉴于罗沙司他和罗特西普改善贫血的作用机制不同，我们设计并实施了本项单中心前瞻性随机对照研究，旨在比较罗沙司他联合罗特西普与罗特西普单药对于难治性MDS-RS的疗效和安全性。

## 病例与方法

一、研究人群

本研究为单中心前瞻性随机对照研究。病例均来自中国医学科学院北京协和医院血液内科。

纳入标准：①年龄≥18岁；②根据2022年WHO标准[10]诊断为MDS-RS；③修订的国际预后积分系统（IPSS-R）危险度分类为极低危、低危或中危；④入组时存在贫血（HGB<100 g/L）或需定期输注红细胞（≥2 U/8周）；⑤足量rhEPO治疗3个月无明显疗效，或预计rhEPO疗效不佳（既往未接受过rhEPO治疗，但内源性EPO>500 IU/L）；⑥入组前，停用既往治疗贫血药物至少2个月；⑦既往未接受过罗沙司他或罗特西普治疗。

排除标准：①合并活动性感染或其他恶性肿瘤；②明显肝肾功能异常（肝肾功能指标超过正常范围2倍）或重要脏器功能异常；③合并骨髓纤维化；④妊娠期或哺乳期女性，或近期有生育需求者；⑤对罗特西普、赋形剂或聚山梨酯80过敏。

基线评估内容包括社会人口学资料、病史、既往输血情况、血常规（含网织红细胞）、生化（肝、肾、甲状腺功能，铁蛋白、转铁蛋白、转铁蛋白饱和度，叶酸，维生素B12）、HAV、HBV、HCV、EBV、CMV、HIV和细小病毒B19，妊娠试验（有生育潜力的妇女尿HCG）、EPO水平、凝血功能、直接抗人球蛋白试验（DAT）、X线胸片、超声心动、腹部超声、骨髓穿刺、活检、染色体、MDS相关基因检测等。

本研究方案（K3697）得到北京协和医院医学伦理委员会审查并批准（I-23PJ1110）。所有患者均签署知情同意书。

二、治疗方案

患者入组后，按照1∶2比例随机分配接受罗沙司他联合罗特西普（以下简称联合用药组）或罗特西普单药治疗（以下简称单药组）。罗特西普初始剂量为1.0 mg/kg每3周1次皮下注射。若使用罗特西普6周后仍疗效不佳，单次剂量加至1.33 mg/kg，后续根据疗效调整剂量（最高1.75 mg/kg）。罗沙司他治疗剂量为150 mg隔日1次。随访期间HGB低于60 g/L时予以红细胞输注。用药12周后进行评估，若HGB≥120 g/L停止用药，HGB<120 g/L时继续使用至出现不可接受的不良反应、疾病进展、撤回同意或达到停药标准。

三、研究终点

主要研究终点为治疗12周时红系反应：输血负担<4 U/8周的患者，HGB较基线增加≥15 g/L；输血负担≥4 U/8周的患者，红细胞输注减少至少4 U/8周[11]。

次要研究终点包括治疗24周时红系反应、治疗12周内脱离输血时间≥8周（仅限需红细胞输注的患者）。其他次要研究终点包括治疗12或24周内脱离输血时间≥12周或16周（仅限需红细胞输注的患者）、治疗12周和24周时输血负担降低水平、HGB变化（输血依赖患者采用输血前HGB水平），治疗12周和24周时白细胞计数、血小板计数、中性粒细胞和血清铁蛋白水平变化以及安全性分析。

四、不良事件评估

参照美国国立癌症研究所的不良事件常用术语标准（CTCAE）第5版定义不良事件。

五、随访方法

治疗前12周，每4周对患者进行1次门诊随访；治疗满12周后，每12周对患者进行1次门诊随访。随访时记录患者症状、治疗相关的不良事件、输血量，进行体格检查，检测血常规（含网织红细胞）、生化（肝肾功能、铁蛋白）。每24周复查骨穿、活检、染色体，MDS相关基因检测，观察疗效及安全性、输血依赖脱离情况。本研究随访截止时间为2024年7月31日。

六、统计学处理

根据功效计算结果，假设联合用药组反应率70％，单药组反应率30％，检验功效为80％，所需样本量为54例患者（联合用药组18例，单药组36例）。此计算结果基于单侧α值0.05，使用汇总方差估计的统计数据，并假定失访率为10％。

所有疗效分析均为意向性分析。描述性统计中，分类变量用“频数（百分比）”表示，符合正态分布的连续变量用“均数±标准差”表示，非正态分布的连续变量用“*M*（*Q*1，*Q*3）”表示。分类变量比较使用卡方检验或Fisher精确检验，符合正态分布的连续变量比较使用*t*检验，非正态分布的连续变量比较使用非参数检验。*P*<0.05被认为差异有统计学意义。

## 结果

一、基线特征

2022年7月1日至2024年5月31日期间，共计入组56例患者，其中1例患者入组时间未满12周，5例患者在治疗开始后12周内失访，2例患者退出研究。最终联合用药组16例、单药组32例患者纳入分析。其中23例患者随访超过24周。

两组患者基线特征差异无统计学意义（表1）。输血依赖方面，联合用药组、单药组分别有18.8％（3/16）、18.8％（6/32）的患者基线红细胞输注量≥4 U/8周，6.2％（1/16）、18.8％（6/32）的患者基线红细胞输注量<4 U/8周，其余患者为非输血依赖。既往接受过来那度胺、去甲基化药物、环孢素A治疗的患者占比分别为37.5％（18/48）、14.6％（7/48）、39.6％（19/48）。

**表1:** 接受罗特西普+罗沙司他（联合用药组）和罗特西普单药（单药组）治疗难治骨髓增生异常肿瘤（MDS）伴环状铁粒幼细胞患者的主要基线特征

特征	联合用药组（16例）	单药组（32例）	统计量	*P*值
年龄（岁，*x*±*s*）	65.6±10.3	64.0±11.2	−0.481	0.64
男性［例（％）］	10（62.5）	20（62.5）	0.000	1.00
诊断MDS至入组时间［月，*M*（*Q*1，*Q*3）］	14.3（13.0,31.3）	12.0（3.8,32.4）	210	0.31
IPSS-R危险度分类［例（％）］			0.011	0.92
低危	9（56.2）	20（62.5）		
中危	7（43.8）	12（37.5）		
血清EPO［例（％）］			0.857	0.36
≤ 500 IU/L	5（31.2）	16（50.0）		
> 500 IU/L	11（68.8）	16（50.0）		
SF3B1突变阳性［例（％）］	11（68.8）	20（62.5）	0.011	0.92
输血依赖［例（％）］			1.393	0.50
非输血依赖	12（75.0）	20（62.5）		
<4 U/8周	1（6.2）	6（18.8）		
≥4 U/8周	3（18.8）	6（18.8）		
输血前HGB（g/L，*x*±*s*）	71.7±17.7	72.2±18.8	0.085	0.93
WBC［×10^9^/L，*M*（*Q*1,*Q*3）］	3.38（2.47,4.40）	3.16（2.44,4.56）	244	0.79
PLT［×10^9^/L，*M*（*Q*1,*Q*3）］	173（108,236）	163（93,220）	229.5	0.56
铁蛋白［µg/L，*M*（*Q*1,*Q*3）］	768（386,1424）	942（461,1549）	271	0.74
网织红细胞绝对值［×10^9^/L，*M*（*Q*1,*Q*3）］	58.4（30.0,67.8）	36.5（10.7,51.6）	22	0.14
既往治疗［例（％）］				
rhEPO	14（87.5）	27（84.4）	0	1.00
来那度胺	4（25.0）	14（43.8）	0.900	0.34
去甲基化药物	3（18.8）	4（12.5）	0.021	0.67
环孢素A	9（56.2）	10（31.2）	1.840	0.18

**注** IPSS-R：修订的国际预后积分系统；rhEPO：重组人促红细胞生成素

二、治疗反应

治疗12周时，联合用药组、单药组分别有37.5％（6/16）、34.4％（11/32）的患者获得红系反应（*P*＝1.00）；输血依赖的患者中，联合用药组、单药组分别有75.0％（3/4）、58.3％（7/12）的患者脱离输血依赖时间≥8周（*P*＝1.00），50％（2/4）、41.7％（5/12）的患者脱离输血≥12周（*P*＝1.00）。治疗24周时，联合用药组、单药组分别有42.9％（3/7）、43.8％（7/16）的患者获得红系反应（*P*＝1.00），联合用药组1例患者完全脱离输血，单药组6例患者中4例（66.7％）脱离输血依赖≥12周，3例（50.0％）脱离输血依赖≥16周。

治疗12、24周时，比较联合用药组、单药组的血小板计数、白细胞计数、中性粒细胞计数及铁蛋白水平较基线的升高值，差异均无统计学意义（*P*>0.05）。在治疗有效组中，上述数值在两组差异亦无统计学意义（*P*>0.05，表2）。

**表2:** 接受罗特西普+罗沙司他（联合用药组）和罗特西普单药（单药组）治疗难治骨髓增生异常肿瘤伴环状铁粒幼细胞患者的疗效指标比较

项目	联合用药组（16例）	单药组（32例）	统计量	*P*值
获得红系反应［例数/总例数（％）］				
12周	6/16（37.5）	11/32（34.4）	<0.0000	1.00
24周	3/7（42.9）	7/16（43.8）	<0.000	1.00
脱离输血依赖［例数/总例数（％）］				
≥8周（1～12周）	3/4（75.0）	7/12（58.3）	<0.000	1.00
12周（1～12周）	2/4（50.0）	5/12（41.7）	<0.000	1.00
≥12周（1～24周）	1/1（100.0）	4/6（66.7）	/	/
≥16周（1～24周）	1/1（100.0）	3/6（50.0）	/	/
1～12周其他指标升高值［*M*（*Q*1,*Q*3）］				
总体（48例）				
PLT（×10^9^/L）	−3（−43,23）	−12（−46,4）	228.00	0.54
WBC（×10^9^/L）	0.38（0.00,0.79）	0.26（−0.40,0.89）	231.5.00	0.59
ANC（×10^9^/L）	0.30（−0.21,1.29）	0.28（−0.13,0.57）	230.50	0.58
血清铁蛋白（µg/L）	−16（−351,221）	15（−79,348）	314.00	0.20
12周获得红系反应组（17例）				
PLT（×10^9^/L）	−9（−69,7）	−18（−38,3）	30.00	0.76
WBC（×10^9^/L）	0.25（0.13,0.48）	0.24（−0.23,0.35）	29.00	0.69
ANC（×10^9^/L）	0.14（−0.32,0.21）	0.11（−0.41,0.31）	34.50	0.88
血清铁蛋白（µg/L）	117（−253,379）	13（−214,383）	34.00	0.92
1～24周其他指标升高值［*M*（*Q*1,*Q*3）］				
总体（23例）				
PLT（×10^9^/L）	13（−24,80）	−44（−61,12）	35.00	0.16
WBC（×10^9^/L）	0.72（−0.13,1.11）	0.71（−0.13,1.53）	40.00	0.64
ANC（×10^9^/L）	0.23（0.12,0.35）	0.36（−0.16,0.73）	41.00	0.58
血清铁蛋白（µg/L）	−221（−305,115）	11（−79,167）	61.00	0.55
24周获得红系反应组（10例）				
PLT（×10^9^/L）	145（16,155）	−60（−61,−9）	7.00	0.42
WBC（×10^9^/L）	0.72（0.30,0.92）	0.64（−0.09,1.21）	10.00	0.91
ANC（×10^9^/L）	0.23（0.18,0.41）	−0.10（−0.25,0.29）	5.00	0.21
血清铁蛋白（µg/L）	−221（−243,−2）	10（−288,125）	11.00	0.91

**注** /：例数过少，未进行统计学分析

治疗12周时，联合用药组HGB为（82.1±24.6）g/L，高于基线（10.4±20.7）g/L；单药组为（78.8±27.5）g/L，高于基线（6.6±23.7）g/L，两组患者HGB升幅差异无统计学意义（*P*＝0.59）。治疗24周时，联合用药组HGB为（83.9±24.2）g/L，高于基线（7.3±13.3）g/L；单药组为（80.3±25.6）g/L，高于基线（12.1±19.4）g/L，两组患者HGB升幅差异无统计学意义（*P*＝0.56）。

三、不良反应

共有10例（20.8％）患者在随访过程中报告了治疗相关不良反应，其中联合用药组3例（18.8％），单药组7例（21.9％）（*P*＝0.90）。高尿酸血症和转氨酶升高是最常见的不良反应，发生率均为6.25％。联合用药组出现的不良反应包括高尿酸血症、转氨酶升高、血胆红素升高、呕吐和背部疼痛，发生率均为6.25％（1/16）。单药组出现的不良反应包括高尿酸血症（6.25％，2/32）、转氨酶升高（6.25％，2/32）、血胆红素升高（3.13％，1/32）、呕吐（3.13％，1/32）、乏力（3.13％，1/32）、咳嗽（3.13％，1/32）和水肿（3.13％，1/32）。未发生3级以上的严重治疗相关不良反应，所有患者经过评估后均可继续接受治疗和随访。

## 讨论

既往研究证实，MDS患者贫血的主要原因之一是造血干细胞的SMAD2/3信号过度激活，引起细胞核内转录中介因子1γ下调，进而降低转录因子GATA-1等基因的表达[12]–[14]。GATA-1在多种血细胞分化过程中发挥重要作用，其表达减少可能导致红系祖细胞凋亡，最终使患者出现贫血[15]。罗特西普是一种可溶性受体融合蛋白，其与特定的TGF-β超家族配体结合，抑制这些配体介导的SMAD2/3信号通路，从而促进红系终末分化[13]。另一方面，罗沙司他是一种HIF脯氨酰羟化酶抑制剂，通过稳定HIF来上调多种铁代谢相关蛋白的基因表达，如参与铁吸收的二价金属转运蛋白1、十二指肠细胞色素b，以及转铁蛋白等[16]。因此，罗特西普和罗沙司他改善MDS患者贫血的具体分子机制并不相同。然而，目前尚无临床试验证实两者联合的疗效。由于罗沙司他和罗特西普都是与rhEPO作用机制完全不同的药物，为探索这两种药物联合是否有更好的疗效，我们设计了此前瞻性随机对照临床试验。

PACE-MDS的Ⅱ期临床试验报告63％（32/51）的患者治疗12周后获得红系反应，38％（16/42）的患者脱离输血依赖至少8周；与之相近，MEDALIST Ⅲ期试验结果显示治疗24周后53％（81/153）的患者获得红系反应，38％（58/153）的患者脱离输血依赖达8周[8],[17]。在以往研究中，罗特西普治疗低风险MDS的有效率为18％～60％[9],[18]–[19]。与上述研究相比，本研究两组患者的红系反应率较低（12周联合用药组37.5％，单药组34.4％），而脱离输血依赖率较高（75.0％联合用药组和58.3％单药组的患者脱离输血达8周）。一方面，这可能与研究所纳入患者的异质性有关。在本研究中，分别有37.5％、14.6％、39.6％的患者既往接受过来那度胺、去甲基化药物、环孢素A治疗，而在PACE-MDS研究中仅17％（10/58）的患者使用过来那度胺[17]。MEDALIST则排除了既往接受过来那度胺、去甲基化药物和免疫抑制剂治疗的患者。本研究56.2％的患者基线EPO>500 IU/L，高于PACE-MDS研究（29％）和MEDALIST研究（14％）。本研究脱离输血依赖率高于PACE-MDS和MEDALIST研究，而与国内之前的多中心回顾性罗特西普治疗研究相近（64.3％），这可能与国内外不同的血液储备和输血策略相关[6],[9]。

罗沙司他已被用于治疗慢性肾病引起的贫血，但对于低风险MDS的疗效和安全性，目前国际上仅有一项Ⅲ期临床研究正在进行[20]。在引入阶段，该研究纳入了24例患者（58％为促红细胞生成药物难治性患者），分为3组接受不同剂量的罗沙司他治疗（1.5、2、2.5 mg/kg），结果显示37.5％的患者在治疗28周时脱离输血依赖[6]。与之相比，本研究患者无论联合罗特西普和罗沙司他，还是单药罗特西普，脱离输血依赖的患者占比都高于前述研究，这可能表明罗特西普对于难治低风险MDS的疗效更为显著。

根据疗效对比，本研究发现罗特西普联合罗沙司他治疗方案在主要研究终点（12周红系反应）及次要研究终点（24周红系反应、脱离输血依赖、HGB变化等）上，均未能显示出优于罗特西普单药治疗的统计学差异（*P*>0.05），表明联合用药方案并未产生预期的协同作用。这一结果可能受多重因素影响：①本研究治疗对象为rhEPO难治或基线EPO水平偏高的患者，同时大部分患者经历多线治疗失败，属于高度难治人群，因此其内在疾病特性可能对现有药物及联合方案反应有限，至少在短期内较难见到明显的优势。②罗沙司他在MDS人群中的最佳使用剂量尚未明确，在联合用药方案中进行剂量调整或疗程延长或可影响其疗效。

安全性方面，本研究不良反应发生率两组均与既往国内罗特西普单药的多中心研究相近，而低于罗沙司他单药研究[6],[9]。本研究发生频率相对较高的不良反应为高尿酸血症和转氨酶升高，两组不良反应发生率相近，且均未发生严重不良反应，说明联合治疗并未增加治疗的不良事件发生率。

综上，本研究结果不支持对rhEPO难治的MDS-RS患者，在罗特西普单药治疗基础上常规加用罗沙司他治疗。本研究样本量较小，随访时间相对较短，以上结论尚需进一步验证。
